# Anatomy‐based, patient‐specific VMAT QA using EPID or MLC log files

**DOI:** 10.1120/jacmp.v16i3.5283

**Published:** 2015-05-08

**Authors:** Dewayne L. Defoor, Luis A. Vazquez‐Quino, Panayiotis Mavroidis, Nikos Papanikolaou, Sotirios Stathakis

**Affiliations:** ^1^ Department of Radiation Oncology University of Texas Health Science Center at San Antonio San Antonio TX USA; ^2^ Midsouth Radiation Physics Little Rock AR USA

**Keywords:** IMRT, EPID, QA, MLC log file, VMAT

## Abstract

In this project, we investigated the use of an electronic portal imaging device (EPID), together with the treatment planning system (TPS) and MLC log files, to determine the delivered doses to the patient and evaluate the agreement between the treatment plan and the delivered dose distribution. The QA analysis results are presented for 15 VMAT patients using the EPID measurements, the ScandiDos Delta^4^ dosimeter, and the beam fluence calculated from the multileaf collimator (MLC) log file. EPID fluence images were acquired in continuous acquisition mode for each of the patients and they were processed through an in‐house MATLAB program to create an opening density matrix (ODM), which was used as the input fluence for the dose calculation in the TPS (Pinnacle^3^). The EPID used in this study was the aSi1000 Varian on a Novalis TX linac equipped with high‐definition MLC. The actual MLC positions and gantry angles were retrieved from the MLC log files and the data were used to calculate the delivered dose distributions in Pinnacle. The resulting dose distributions were then compared against the corresponding planned dose distributions using the 3D gamma index with 3 mm/3% passing criteria. The ScandiDos Delta^4^ phantom was also used to measure a 2D dose distribution for all the 15 patients and a 2D gamma was calculated for each patient using the Delta^4^ software. The average 3D gamma using the EPID images was 96.1%±2.2%. The average 3D gamma using the log files was 98.7%±0.5%. The average 2D gamma from the Delta^4^ was 98.1%±2.1%. Our results indicate that the use of the EPID, combined with MLC log files and a TPS, is a viable method for QA of VMAT plans.

PACS numbers: 87.55.Qr

## I. INTRODUCTION

Pretreatment quality assurance of volumetric arc therapy (VMAT) can be performed in a variety of ways, depending on the capabilities of the dosimeter used. For 2D diode array detectors, such as MapCHECK (Sun Nuclear, Melbourne, FL), OCTAVIUS I (PTW, Freiburg, Germany) or MatriXX (IBA, Schwarzenbruck, Germany), the planar dose at isocenter for the entire VMAT plan is commonly measured either with the gantry stationary and the beam orthogonal to the detector or with the aid of a gantry mounting fixture where the detector is attached and is again always orthogonal to the beam.^1^, ^2^, ^3^ While this eliminates any angular dependence, which can be as large as 20%,^2^, ^4^ it also prevents the detection of gantry angle errors. The Delta^4^ (ScandiDos, Uppsala, Sweden) utilizes orthogonal diode detector arrays to provide full coverage of the cross section of any beam direction while measuring dose with the gantry rotating. To eliminate angular dependence, the PTW OCTAVIUS 4D Quality Assurance (QA) system utilizes a motorized cylinder that rotates with the gantry as directed by an inclinometer attached to the gantry. The motorized motion of the detector enforces a perpendicular geometry at all times between the incident beam and the detector plane, resulting in good agreement between measurements and calculations.^(5)^ Although these devices have been widely used for QA measurements, they present their own challenges. Each of these devices requires an accurate setup, which increases the overall time of the test and, if not done correctly, will affect the accuracy of the results. Accuracy is also affected by the number of diodes or ion chambers in the plane of measurement.

There are many publications^6^, ^7^, ^8^, ^9^, ^10^, ^11^, ^12^, ^13^, ^14^, ^15^, ^16^, ^17^, ^18^, ^19^, ^20^ supporting the use of the electronic portal imaging device (EPID) as a QA device. There are commercially available QA systems that utilize the EPID, such as EPIDose (Sun Nuclear) and Portal Dosimetry (Varian Medical Systems, Palo Alto, CA). Although both of these systems use different algorithms for dose comparison, they are both ideal alternatives to the traditional QA systems.^21^, ^22^ The advantages of using EPID for VMAT QAs mainly stem from its simple setup and high resolution of the detector plane (1024×768). Fredh et al.^(23)^ have shown that EPID is more efficient in detecting planning errors than conventional QA devices. Some disadvantages of the EPID as a QA device are the nonwater equivalence of the detector and its inability to detect gantry angle errors through measurements of the fluence.

Dose reconstruction using information from the log files of the multileaf collimator (MLC) of the treatment machine is another useful method for performing a VMAT QA.^24^, ^25^, ^26^, ^27^ Since there are no requirements for physical measurements with this method, all the calculations must be done based on the MLC positions, collimator angle, gantry angle, and cumulative dose per control point that have been recorded in the MLC log file. This method relies on the ability of the machine to accurately record the parameters with which the treatment plan was delivered, and it can be a very efficient and reliable way to perform VMAT QAs.^(26)^

One of the disadvantages of using 2D detector arrays for performing patient specific QAs is that the comparison is performed between a dose plane calculated in the phantom and a single (or two) plane measured with the 2D detector array. In this study, the proposed EPID and MLC log file dose reconstruction methodologies utilize the ability of the TPS to calculate the delivered dose in the patient's anatomy. This dose can then be directly compared against the intended dose distribution of the original treatment plan.

A major advantage of using the TPS for dose calculations is that the quality of the dose distribution can be evaluated in the same software as the initial plan and on the original patient computed tomography (CT) image set. Having the same algorithm for both the original plan and the dose reconstruction removes any ambiguity in the calculations, and any differences in the plan comparison are entirely attributable to machine performance. Furthermore, it is also convenient to evaluate the delivered dose distribution in the same environment that was used to produce the treatment plan in the first place and with which there is already a certain degree of familiarity. By doing so, the user can evaluate the location of the maximum dose points, isodose lines, and cold spots and perform region‐of‐interest statistics, as well as dose‐volume histogram comparisons. This study focuses on the comparison of the three aforementioned QA methods, which are based on physical dose measurements in a phantom, fluence measurements using EPID, and dose calculations using data from MLC log files. The physical dose measurements were only used in comparisons using the gamma index and they were not imported into the TPS. For each patient, the comparisons between the treatment plan and the EPID or MLC log file method were performed using the corresponding dose‐volume histograms (DVH) of the organs and dose distributions. Furthermore, the gamma index was used to evaluate the agreement of the dose distributions reconstructed using the proposed methods against the planned one.

## II. MATERIALS AND METHODS

### A. Clinical cases

Fifteen cancer patients (5 head and neck, 5 lung, and 5 prostate) who were planned and optimized using the Pinnacle^3^ (Philips Radiation Oncology Systems, Fitchburg, WI) treatment planning system (TPS) using two VMAT beams, were used in this study. A list of patients' parameters is shown in Table 1.

**Table 1:** Treatment information for the 15 patients.

*Patient Parameters*			
	*Site*	*MUs*	*Energy*
1	Left Larynx	601	6 MV
2	Oral Cavity	659	6 MV
3	Retromolar	371	6 MV
4	Parotid	277	6 MV
5	Tonsil	349	6 MV
6	Lung	538	6 MV
7	Lung	522	6 MV
8	Lung	668	6 MV
9	Lung	545	6 MV
10	Lung	1092	6 MV
11	Prostate	646	10 MV
12	Prostate	542	10 MV
13	Prostate Bed	516	6 MV
14	Prostate	1322	10 MV
15	Prostate	648	6 MV

### B. Physical measurements

For comparison, physical dose measurements where made using the Delta^4^ on a Novalis TX linac (Varian Medical Systems) equipped with a high definition MLC. The Delta^4^ utilizes two orthogonal detector planes with a total of 1,069 p‐type diode detectors each with a 0.78 mm^2^ active volume enclosed in a cylindrical phantom. The detectors cover a 20 cm×20 cm area and are spaced at 0.5 cm grid in the central 6 cm×6 cm area of the detector planes and at 1 cm intervals outside the central region.

The Delta^4^ software allows the calculation of the 3D dose distribution based on the measured data. This is done by using the planned dose distributions and by interpolating along the ray lines passing through one of the two detector planes. An evaluation of the Delta^4^'s performance by Sadagopan et al.^(28)^ has shown that the interpolated values show good agreement with diode measurements at the interpolated points.

The planned planar dose distribution for each patient was imported into the Delta^4^ software and, after the dose distribution was measured, a 2D gamma was calculated using a global gamma (normalization at 90% of maximum dose) with 3 %/3 mm criteria.

### C. MLC log files

MLC log files contain information on the parameters of the treatment machine and MLC recorded every 50 ms during plan delivery. Information from these files, such as MLC leaf positions, gantry angle, and collimator angle, are integral in reconstructing the dose delivered by the machine. A table of parameters recorded in a VMAT MLC log file is shown in Table 2. A MLC log file was created for each MLC bank of every beam. For example, for a treatment plan that has two beams there are four log files, each beam will have a file for bank A and a file for bank B. An in‐house MATLAB program (MathWorks, Natick, MA) was used to convert the log files, which were recorded during the EPID fluence measurements, into a structure of variables that can be easily accessed and determine the log entries that correspond to beam control points. It should be mentioned that the MLC log files are not affected by the presence or absence of a patient or a phantom in the path of the beam.

**Table 2:** The recorded parameters and their precision based on the MLC log files.

*# MLC Leaves*	*Precision Exact*
Beam On	Binary
Beam Off	Binary
Gantry Angle	$10^{-1}\;\text{degrees}$
Collimator Angle	$10^{-1}\;\text{degrees}$
x1 Jaw Position	$10^{-1}\;\text{mm}$
x2 Jaw Position	$10^{-1}\;\text{mm}$
y1 Jaw Position	$10^{-1}\;\text{mm}$
y2 Jaw Position	$10^{-1}\;\text{mm}$
Carriage Position	$10^{-2}\;\text{mm}$
Leaf Position	$10^{-4}\;\text{mm}$

The locations of the control points were determined by matching the gantry angle and closest MLC shape to that of each control point. All recorded gantry and collimator angles were within half a degree of the corresponding control point. Since Pinnacle does not allow fractional gantry and collimator angles as inputs, only the actual MLC leaf positions recorded in the MLC log file were substituted in place of the planned parameters in the patient plan file and the dose was recalculated in the TPS using the MLC log file data. The dose was then exported to VeriSoft (PTW) and a 3D gamma was calculated using 3 mm/3% criteria.

### D. EPID

The EPID used in this study was the aSi1000 (PortalVision, Varian Medical Systems) on a Novalis TX linac equipped with high definition MLC. The aSi1000 has an amorphous silicon detector with an active imaging area of $40\times 30\;{\text{cm}}^{2}$ and a 1024×768 array of pixels. With a center‐to‐center pixel spacing of 0.0392 cm, the EPID has a much higher resolution than the Delta^4^.

Images for each beam were captured in continuous acquisition mode with the EPID located at isocenter without a phantom or patient in the beam. In continuous acquisition mode the EPID captures eight images per second and the eight images are averaged to produce an image approximately every 4°. A calibration image was taken at the time of measurement to obtain an accurate intensity to MU conversion. A MATLAB in‐house software program (MU‐EPID) was used to convert the fluence map captured by the EPID into an Opening Density Matrix (ODM) for import into Pinnacle.^(29)^

**Figure 1:**
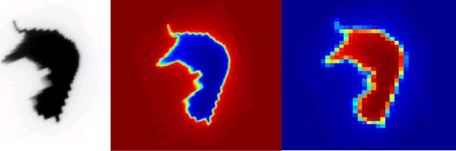
A 758×1024 pixel EPID image (left) is converted to a MATLAB image (middle) and then to a 101×101 pixel ODM (right).

The MU‐EPID software applies a correction matrix to each image to account for the spatial variation in the EPID response. The correction matrix was acquired by comparing dose planes in a water phantom calculated with a starting fluence measured by the EPID to a dose plane calculated with Pinnacle's starting fluence. After the correction matrix was applied, the image was normalized and resized in order to be imported as a valid fluence into Pinnacle. An example of the image taken by the EPID and the resulting ODM is shown in Fig. 1. The ODMs were imported into Pinnacle using a script which creates a beam for each image with the parameters recorded in the DICOM image obtained from the EPID. The dose distributions were then calculated using the ODM as a starting fluence, the calculated dose was exported to PTW VeriSoft, and a 3D gamma index was calculated using 3 mm distance to agreement (DTA) and 3% dose difference criteria.

## III. RESULTS

### A. Evaluation in Pinnacle

#### A.1 EPID

On average, the deviations of dose received by 2% of the PTV ($D_{2\%}$) and dose received by 98% of the PTV ($D_{98\%}$) over all the patients were 2.46% and 5.97%, respectively. The mean dose to the PTV for all the patients was within 1% when compared to the treatment plan. The doses to the OARs over all the patients were within 4.30% compared to those of the treatment plan. Deviations from the approved plan by treatment site are shown in 3, 5. All the deviation percentages were calculated using the absolute differences on planned and calculated values.

**Table 3:** Deviation from planned values of dose received by 2% of the PTV ($D_{2\%}$), dose received by 98% of the PTV ($D_{98\%}$), mean PTV dose, and dose to the organs at risk for the head and neck patients.

*% Deviation From Approved Plan — H/N*					
	$D_{2\%}$	$D_{98\%}$	*Mean PTV*	*Brainstem*	*Spinal Cord*
EPID	2.7%	5.8%	1.2%	9%	6.4%
MLC Logs	1.7%	0.9%	0.6%	1.4%	1.4%

**Table 5:** Deviation from planned values of dose received by 2% of the PTV ($D_{2\%}$), dose received by 98% of the PTV ($D_{98\%}$), mean PTV dose, and dose to the organs at risk for the prostate patients.

*% Deviation From Approved Plan — Prostate*					
	$D_{2\%}$	$D_{98\%}$	*Mean PTV*	*Rectum*	*Bladder*
EPID	2.7%	5.7%	1.0%	0.5%	1.5%
MLC Logs	0.1%	1.2%	0.1%	0.2%	0.6%

**Figure 2:**
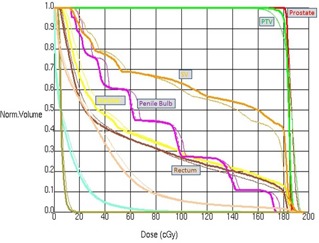
The DVH calculated by Pinnacle for the treatment plan (solid), EPID images (dashed), and MLC log file (thin dashed) for a representative patient. The MLC log file lines are hidden behind the treatment plan lines.

**Figure 3:**
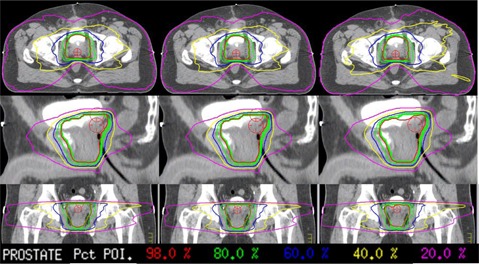
Dose distribution comparisons of the treatment plan (left), MLC log file data (middle), and EPID ODM (right) for a representative patient.

Figure 2 shows a dose‐volume histogram comparison of the three methods for a typical patient in this study, and Fig. 3 shows the dose distributions for the same patient. The dose‐volume histogram shows very good agreement in the high‐dose regions, with minimal differences in low‐dose region. This pattern is representative of all the patients in the study and in some cases the DVH lines are completely overlapping. Geometrically, the dose distribution agrees with the planned distribution. The shapes of the isodose lines of the delivered dose distributions match those of the treatment plan well in all the views.

### B. MLC log files

$D_{2\%}$ and $D_{98\%}$ of the PTV over all the patients is on average within 0.6% and 1%, respectively. The mean doses to the PTV over all the patients are within 0.35% compared to those of the treatment plan (3, 5, 4). The doses to the OARs are on average within 2.4% of the treatment plan.

**Table 4:** Deviation from planned values of dose received by 2% of the PTV ($D_{2\%}$), dose received by 98% of the PTV ($D_{98\%}$), mean PTV dose, and dose to the organs at risk for the lung patients.

*% Deviation From Approved Plan — Lung*					
	$D_{2\%}$	$D_{98\%}$	*Mean PTV*	*Total Lung*	*Esophagus*
EPID	2.0%	6.4%	0.6%	1.5%	6.4%
MLC Logs	0.6%	0.6%	0.4%	1.2%	5.2%

The DVHs of the MLC log file method for a typical patient are indistinguishable from those of the treatment plan and this is consistent over all the patients. The deviations in the MLC positions and dose fraction are so small that dose distribution is minimally affected.

For this method, the delivered dose distribution agrees very well with the planned distribution, and the shapes of the isodose lines match the approved plan very well in all the views. There is very little variance in the dose distributions, which was expected with such small MLC deviations.

### C. MLC log file statistics

**Figure 4:**
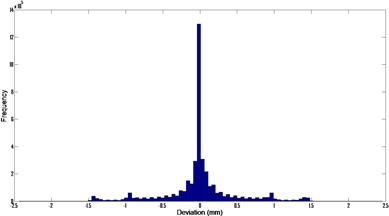
Histogram of the MLC Deviations for the 31 VMAT beams (15 patients).

**Figure 5:**
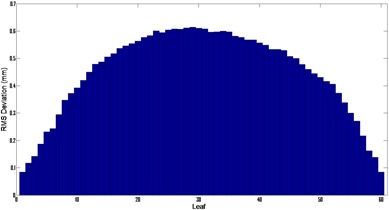
The RMS error by leaf for all the VMAT beams

A histogram of the MLC deviations is shown in Fig. 4 with a mean RMS deviation of 0.27 mm. MLC positions deviated from the planned positions by an average of 2.3%. Figure 5 shows the mean RMS leaf deviation for all the beams. The outer leaves generally move the least and, therefore, have the smallest deviation. All the statistics were computed using all the control points for all the patients.

### D. 3D gamma

A 3D gamma index was calculated for all the patients for both the EPID and MLC log file methods, whereas a 2D gamma index was calculated for the Delta^4^. A summary of the gamma index values is shown in Table 6. Not surprisingly, the MLC log file method resulted in the highest gamma index passing rate percentages with the least variation, while the EPID and Delta^4^ showed similar results. Although the EPID method calculates a gamma in 3D, its results on a 2D plane are similar to those by Delta^4^.

**Table 6:** Gamma percentages for all three methods compared with the approved plan.

*Gamma %*																
	*1*	*2*	*3*	*4*	*5*	*6*	*7*	*8*	*9*	*10*	*11*	*12*	*13*	*14*	*15*	*Average*
EPID MLC	95.9	97.6	98.5	94.6	96.8	97.8	98.4	91.0	97.2	96.8	92.4	93.7	95.3	96.9	98.0	96.1±2.2
Logs	99.1	98.3	98.7	99.1	98.5	98.9	99.0	98.6	98.8	99	97.8	97.9	98.6	98.5	99.6	98.7±0.5
Delta^4^	99.5	99.0	91.9	99.2	99.9	99.5	98.1	96.9	98.1	95.0	99.3	100	99.5	97.2	97.9	98.1±2.1

## IV. DISCUSSION

The results of this study support the use of our proposed alternative QA methods for pretreatment verification. The use of the TPS as a dose calculation tool, as well as a means to visualize the delivered dose distribution on patient anatomy, has major advantages over the traditional QA systems.

All patients in this study show good agreement with the approved plan for both the EPID and MLC log file methods (apart from two of the patients where the EPID values were considerably lower than those of the Delta^4^ and MLC log file methods, possibly due to the overresponse of EPID to low energy scatter from the patient in cases involving small fields). The dose distributions show similar maximum dose locations and magnitudes, as well as shape of the isodose lines. Dose‐volume histograms have similar dose falloff in the target region for all the patients in our study. The 3D gamma percentage values are highest for the MLC log file method, which has a higher inherent agreement with the treatment plan, whereas the 3D gamma values of the EPID are as also very comparable. The EPID performed similarly to the Delta^4^ regarding the 2D gamma results. The EPID results agree with previous work by Vazquez Quinoet al.^(29)^

The EPID method produces an accurate representation of the photon fluence from the gantry head, which translates to an accurate calculated dose distribution in Pinnacle. The efficiency of this method is complimented by its accuracy. The ability to perform dose calculations using the TPS eliminates potential differences stemming from the use of different calculation algorithms and it has the advantage of displaying the reconstructed dose on the true patient anatomy. Furthermore, it has been recently reported in the literature that relying solely on gamma analysis can allow systematic errors to go undetected.^(30)^

For the MLC log file method, the measurements and results that were acquired in the present study regarding the DVHs, dose distributions, and MLC deviations between the delivered, and planned treatments are in agreement with previous work by Schreibmann et al.^(26)^ and Agnew et al.^(24)^ Similar to the EPID method, the MLC log file method requires very little time at the treatment machine and the data can be gathered by a therapist if necessary.

## V. CONCLUSIONS

The EPID method produced results comparable to the results of the Delta^4^, which is our established QA method. Use of the MLC log file information revealed little deviation of the delivered plans from the approved plans. While using the gamma index as a comparison metric can be valuable in quantifying the agreement between two dose distributions in a phantom, no information is revealed about the clinical effect the differences between the delivered and calculated dose distribution might have to the patient. Furthermore, planar dose comparison between calculations and measurements using phantom geometry omits information related to patient anatomy. For this reason, we recommend extending the patient‐specific IMRT QA criteria to include differences in the DVH and dose distribution in the patient anatomy. The ability to perform an accurate patient‐specific VMAT QA in which the effects of the differences in dose distributions can be quantified on patient anatomy by merely utilizing basic clinical resources provides the opportunity for any clinic to improve their QA process at no monetary cost. The only investment will be the time to create a link between the EPID/MLC log files and the TPS. Furthermore, the ability to combine these methods allows customization to the specific needs of each clinic.

## Supporting information

Supplementary Material

## References

[1] Jursinic PA , Nelms BE . A 2‐D diode array and analysis software for verification of intensity modulated radiation therapy delivery. Med Phys. 2003;30(5):870–79. 12772995 10.1118/1.1567831

[2] Jursinic PA , Sharma R , Reuter J . MapCHECK used for rotational IMRT measurements: step‐and‐shoot, TomoTherapy, RapidArc. Med Phys. 2010;37(6):2837–46. 20632595 10.1118/1.3431994

[3] Nelms BE and Simon JA . A survey on planar IMRT QA analysis. J Appl Clin Med Phys. 2007;8(3):2448. 17712302 10.1120/jacmp.v8i3.2448 PMC5722600

[4] Rinaldin G , Perna L , Agnello G , et al. Quality assurance of Rapid Arc treatments: performances and pre‐clinical verifications of a planar detector (MapCHECK2). Phys Med. 2014;30(2):184–90. 23751395 10.1016/j.ejmp.2013.05.004

[5] Stathakis S , Myers P , Esquivel C , Mavroidis P , Papanikolaou N . Characterization of a novel 2D array dosimeter for patient‐specific quality assurance with volumetric arc therapy. Med Phys. 2013;40(7):071731. 23822435 10.1118/1.4812415

[6] Hussein M , Rowshanfarzad P , Ebert MA , Nisbet A , Clark CH . A comparison of the gamma index analysis in various commercial IMRT/VMAT QA systems. Radiother Oncol. 2013;109(3):370–76. 24100148 10.1016/j.radonc.2013.08.048

[7] Liu B , Adamson J , Rodrigues A , Zhou F , Yin FF , Wu Q . A novel technique for VMAT QA with EPID in cine mode on a Varian TrueBeam linac. Phys Med Biol. 2013;58(19):6683–700. 24018655 10.1088/0031-9155/58/19/6683

[8] Van Zijtveld M , Dirkx ML , de Boer HC , Heijmen BJ . Dosimetric pre‐treatment verification of IMRT using an EPID; clinical experience. Radiother Oncol. 2006;81(2):168–75. 17055604 10.1016/j.radonc.2006.09.008

[9] Vieira SC , Bolt RA , Dirkx ML , Visser AG , Heijmen BJ . Fast, daily linac verification for segmented IMRT using electronic portal imaging. Radiother Oncol. 2006;80(1):86–92. 16854483 10.1016/j.radonc.2006.06.010

[10] Greer PB and Popescu CC . Dosimetric properties of an amorphous silicon electronic portal imaging device for verification of dynamic intensity modulated radiation therapy. Med Phys. 2003;30(7):1618–27. 12906179 10.1118/1.1582469

[11] Lin MH , Li J , Wang L , et al. 4D patient dose reconstruction using online measured EPID cine images for lung SBRT treatment validation. Med Phys. 2012;39(10):5949–58. 23039633 10.1118/1.4748505

[12] Nijsten SM , Mijnheer BJ , Dekker AL , Lambin P , Minken AW . Routine individualised patient dosimetry using electronic portal imaging devices. Radiother Oncol. 2007;83(1):65–75. 17383761 10.1016/j.radonc.2007.03.003

[13] Nijsten SM , Minken AW , Lambin P , Bruinvis IA . Verification of treatment parameter transfer by means of electronic portal dosimetry. Med Phys. 2004;31(2):341–47. 15000620 10.1118/1.1640972

[14] Partridge M , Ebert M , Hesse BM . IMRT verification by three‐dimensional dose reconstruction from portal beam measurements. Med Phys. 2002;29(8):1847–58. 12201432 10.1118/1.1494988

[15] Renner WD , Norton K , Holmes T . A method for deconvolution of integrated electronic portal images to obtain incident fluence for dose reconstruction. J Appl Clin Med Phys. 2005;6(4):22–39. 10.1120/jacmp.v6i4.2104 PMC5723452 16421498

[16] Renner WD , Sarfaraz M , Earl MA , Yu CX . A dose delivery verification method for conventional and intensity‐modulated radiation therapy using measured field fluence distributions. Med Phys. 2003;30(11):2996–3005. 14655947 10.1118/1.1610771

[17] Sharma DS , Mhatre V , Heigrujam M , Talapatra K , Mallik S . Portal dosimetry for pretreatment verification of IMRT plan: a comparison with 2D ion chamber array. J Appl Clin Med Phys. 2010;11(4):3268. 21081884 10.1120/jacmp.v11i4.3268 PMC5720403

[18] Steciw S , Rathee S , Warkentin B . Modulation factors calculated with an EPID‐derived MLC fluence model to streamline IMRT/VMAT second checks. J Appl Clin Med Phys. 2013;14(6):4274. 24257271 10.1120/jacmp.v14i6.4274 PMC5714641

[19] Steciw S , Warkentin B , Rathee S , Fallone BG . Three‐dimensional IMRT verification with a flat‐panel EPID. Med Phys. 2005;32(2):600–12. 15789607 10.1118/1.1843471

[20] Warkentin B , Steciw S , Rathee S , Fallone BG . Dosimetric IMRT verification with a flat‐panel EPID. Med Phys. 2003;30(12):3143–55. 14713081 10.1118/1.1625440

[21] Bailey DW , Kumaraswamy L , Bakhtiari M , Malhotra HK , Podgorsak MB . EPID dosimetry for pretreatment quality assurance with two commercial systems. J Appl Clin Med Phys. 2012;13(4):3736. 22766944 10.1120/jacmp.v13i4.3736 PMC5716510

[22] Varatharaj C , Moretti E , Ravikumar M , Malisan MR , Supe SS , Padovani R . Implementation and validation of a commercial portal dosimetry software for intensity‐modulated radiation therapy pre‐treatment verification. J Med Phys. 2010;35(4):189–96. 21170182 10.4103/0971-6203.71758 PMC2990112

[23] Fredh A , Scherman JB , Fog LS , Munck af Rosenschold P . Patient QA systems for rotational radiation therapy: a comparative experimental study with intentional errors. Med Phys. 2013;40(3):031716. 23464311 10.1118/1.4788645

[24] Agnew CE , King RB , Hounsell AR , McGarry CK . Implementation of phantom‐less IMRT delivery verification using Varian DynaLog files and R/V output. Phys Med Biol. 2012;57(21):6761–77. 23032423 10.1088/0031-9155/57/21/6761

[25] Dinesh Kumar M , Thirumavalavan N , Venugopal Krishna D , Babaiah M . QA of intensity‐modulated beams using dynamic MLC log files. J Med Phys. 2006;31(1):36–41. 21206638 10.4103/0971-6203.25668 PMC3003892

[26] Schreibmann E , Dhabaan A , Elder E , Fox T . Patient‐specific quality assurance method for VMAT treatment delivery. Med Phys. 2009;36(10):4530–35. 19928084 10.1118/1.3213085

[27] Teke T , Bergman AM , Kwa W , Gill B , Duzenli C , Popescu IA . Monte Carlo based, patient‐specific RapidArc QA using Linac log files. Med Phys. 2010;37(1):116–23. 20175472 10.1118/1.3266821

[28] Sadagopan R , Bencomo JA , Martin RL , Nisson G , Matzen T , Balter PA . Characterization and clinical evaluation of a novel IMRT quality assurance system. J Appl Clin Med Phys. 2009;10(2):2928. 19458595 10.1120/jacmp.v10i2.2928 PMC5720456

[29] Vazquez Quino LA , Chen X , Fitzpatrick M , et al. Patient specific pre‐treatment QA verification using an EPID approach. Technol Cancer Res Treat. 2014;13(1):1–10. 23819492 10.7785/tcrt.2012.500351

[30] Nelms BE , Chan MF , Jarry G , et al. Evaluating IMRT and VMAT dose accuracy: practical examples of failure to detect systematic errors when applying a commonly used metric and action levels. Med Phys. 2013;40(11):111722. 24320430 10.1118/1.4826166 PMC8353583

