# Supplementary figures and images for: Anatomy‐based, patient‐specific VMAT QA using EPID or MLC log files
Source: J Appl Clin Med Phys. 2015 May 8;16(3):206–15. doi: 10.1120/jacmp.v16i3.5283 (PMC5690143; doi:10.1120/jacmp.v16i3.5283)

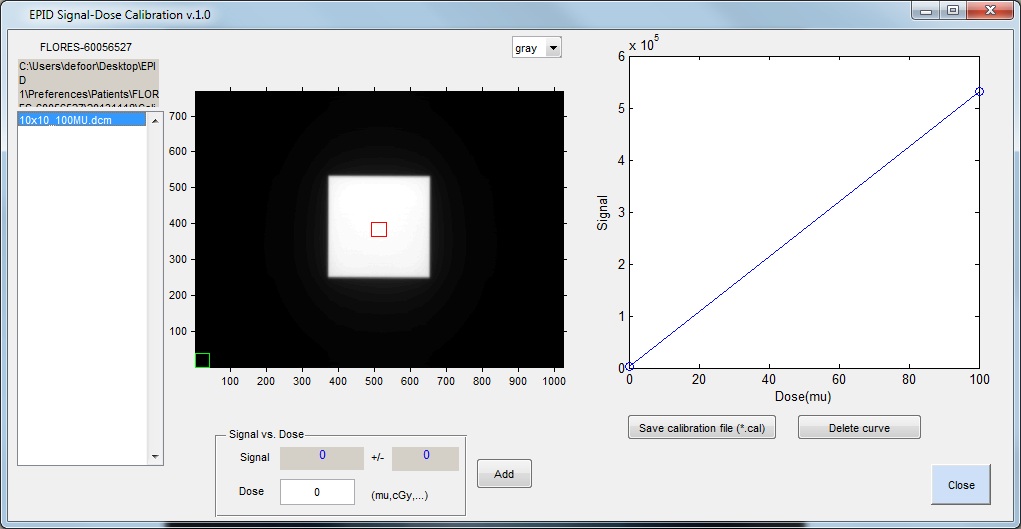

Supplement: Supplementary file 1 — Supplementary Material [file ACM2-16-206-s001.jpg]
